# Improved hypopharyngeal visibility and early cancer detection using the modified Killian method during esophagogastroduodenoscopy in patients at high risk of head and neck cancer

**DOI:** 10.1007/s10388-026-01220-4

**Published:** 2026-06-02

**Authors:** Yuki Yoshida, Yohei Ikenoyama, Toru Ogura, Hiroto Suzuki, Aiji Hattori, Yuhei Umeda, Akina Shigefuku, Yasuko Fujiwara, Misaki Nakamura, Yasuhiko Hamada, Noriyuki Horiki, Hayato Nakagawa

**Affiliations:** 1 Department of Gastroenterology and Hepatology, Mie University Graduate School of Medicine, Tsu, Mie Japan; 2 Clinical Research Support Center, Mie University Hospital, Tsu, Mie Japan

**Keywords:** Pharyngeal neoplasms, Early detection of cancer, Esophagogastroduodenoscopy, Valsalva maneuver, Modified Killian method

## Abstract

**Background:**

Patients with a history of esophageal squamous cell carcinoma or pharyngeal cancer are at high risk of laryngopharyngeal cancers, particularly in the hypopharynx. However, adequate hypopharyngeal visualization during esophagogastroduodenoscopy is challenging because of anatomical constraints. We evaluated the utility of the Modified Killian method for hypopharyngeal visualization during esophagogastroduodenoscopy in high-risk patients.

**Methods:**

The Modified Killian method had previously been used at our institution for high-risk patients with esophageal and head and neck cancers. In this retrospective comparative study, data were collected from 45 high-risk patients who underwent pharyngeal examination using the Modified Killian method after the conventional method during a single esophagogastroduodenoscopy session. The primary endpoint was the hypopharyngeal visibility score (scale 1–5). Secondary endpoints included visibility of other pharyngeal areas, procedure time, lesion detection, and adverse events.

**Results:**

The Modified Killian method without the Valsalva maneuver yielded higher hypopharyngeal visibility scores than the conventional method (median [interquartile range, IQR]: 2.0 [2.0–4.0] vs. 1.0 [1.0–2.0]; *p* < 0.001). The Modified Killian method further improved visibility (median [IQR]: 4.0 [3.0–5.0]; *p* < 0.001 vs. conventional). No significant differences were observed in visibility of the oropharynx or vallecula. The procedure time was longer for the Modified Killian method (237 vs. 134 s; *p* < 0.001). Three intraepithelial hypopharyngeal carcinomas missed by the conventional method were detected with the Modified Killian method. No adverse events occurred.

**Conclusions:**

The Modified Killian method, particularly its positional component alone, improves hypopharynx visualization and may contribute to early cancer detection without compromising observation of other pharyngeal areas.

**Supplementary Information:**

The online version contains supplementary material available at 10.1007/s10388-026-01220-4.

## Introduction

Laryngopharyngeal cancer is a primary cause of death in patients with head and neck squamous cell carcinoma (HNSCC), accounting for approximately 2.6% of all cancer-related deaths [1]. Its prognosis is often poor due to diagnosis at advanced stages. Therefore, early detection via esophagogastroduodenoscopy (EGD) is crucial for reducing mortality [2]. HNSCC, particularly in the hypopharynx, frequently co-occurs with esophageal squamous cell carcinoma (ESCC) due to “field cancerization [3]”. Chronic exposure to carcinogens, such as alcohol and tobacco, predisposes the upper aerodigestive tract to multiple carcinomas [4]. Given the high cumulative incidence of HNSCC in patients with ESCC, endoscopists carefully inspect the pharynx and larynx [5]. However, despite advances such as narrow-band imaging (NBI), the hypopharynx remains difficult to visualize due to anatomical constraints, often resulting in tumor detection at more advanced stages [6]. Conventional techniques such as phonation or the Valsalva maneuver have variable effectiveness [7]. Recent otolaryngology reports indicate that the Modified Killian (MK) method—combining head torsion with forward neck flexion—provides a broader, clearer hypopharynx view than do the conventional methods [8–10]. However, its efficacy in gastrointestinal endoscopy is relatively unknown. Herein, we aimed to retrospectively evaluate the contribution of the MK method to the expansion of the hypopharyngeal field of view during EGD and provide evidence for its adoption as a standard technique for high-risk patients.

## Methods

### Study design and patient selection

In this single-center retrospective comparative study, we evaluated conventional observation and the MK method for pharyngeal examination. Since the MK method had already been incorporated into routine clinical practice at our institution for high-risk patients, we retrospectively used this existing workflow as a de facto comparative protocol, with conventional observation consistently performed first, followed by the MK method.

Patients with a current or past ESCC diagnosis, or a history of pharyngeal cancer treated with methods such as endoscopic submucosal dissection (ESD) or chemoradiotherapy, were considered high-risk patients. At our institution, the MK method is routinely attempted during surveillance endoscopy for all patients identified as high risk for laryngopharyngeal cancer. During this study, we analyzed the data from 45 consecutive patients from September 2024 to August 2025 who underwent pharyngeal examination using the MK method following conventional observation. No patients were arbitrarily excluded. Patients were excluded only if they had undergone prior surgical resection of the pharynx, had an Eastern Cooperative Oncology Group performance status > 2, had dementia, or had a psychiatric disorder preventing them from following instructions, or if they declined to participate via the opt-out process. All patients were informed before the procedure that the examination could be discontinued at their request if significant discomfort occurred. No additional sedation or analgesics were administered for pharyngeal observation. The study was approved by the Mie University Hospital ethics committee (IRB No. H2021-116). The requirement for individual informed consent was waived, and an opt-out approach was applied.

### The MK method

The MK method begins by placing the patient in the Killian position (neck flexed, chin tucked). Further neck flexion creates the MK position. Performing the Valsalva maneuver while turning the head in this position provides a wider endoscopic field of view. Online Resource 1 demonstrates the procedure. The conventional method (observation in the left lateral decubitus position using a transnasal endoscope) was used for comparison.

### Examination procedure

Three gastrointestinal endoscopists certified by the Japan Gastroenterological Endoscopy Society performed all procedures. Pharyngeal observations were performed using a transnasal endoscope (GIF-1200 N; Olympus Corporation) combined with a video endoscope system (EVIS X1; Olympus Corporation). Before the procedure, 2 mL of 2% viscous lidocaine was administered into the more patent nostril to provide local pharyngeal anesthesia.

The following procedure was followed for each patient:

Step 1: Each patient first underwent conventional observation in the left lateral decubitus position.

Step 2: The patient was then repositioned to a seated position, and observation was performed using the MK method.

Step 3: During the MK method, hypopharyngeal images were captured before and after the Valsalva maneuver.

With the exception of the Valsalva maneuver, no additional patient-assisted maneuvers, such as phonation or other techniques aimed at improving hypopharyngeal visualization, were employed during either the conventional method or the MK method.

Following pharyngeal observation, the patient was repositioned to the left lateral decubitus position, and the esophagus, stomach, and duodenum were examined using an oral endoscope with magnifying capability. This sequential workflow is practical and readily integrable into routine endoscopic practice. The examiners began at the oropharynx and observed and captured images of the oropharynx [(i) posterior wall (PW), (ii) left lateral wall, and (iii) right lateral wall], vallecula, and posterior hypopharyngeal wall and bilateral pyriform sinuses. Observations were conducted in white-light mode to compare visibility; image-enhanced endoscopy (NBI) was used only if a lesion was suspected. The Valsalva maneuver is generally desirable for hypopharyngeal observations [11]; however, we did not use it during conventional observation as it can induce coughing and gag reflexes, possibly interfering with subsequent observations. Images captured before the Valsalva maneuver during the MK method were defined as “the MK method without the Valsalva maneuver.” Thus, the hypopharynx was observed under three conditions: the conventional method, the MK method without the Valsalva maneuver, and the MK method.

### Image handling and anonymization

**Fig. 1:**
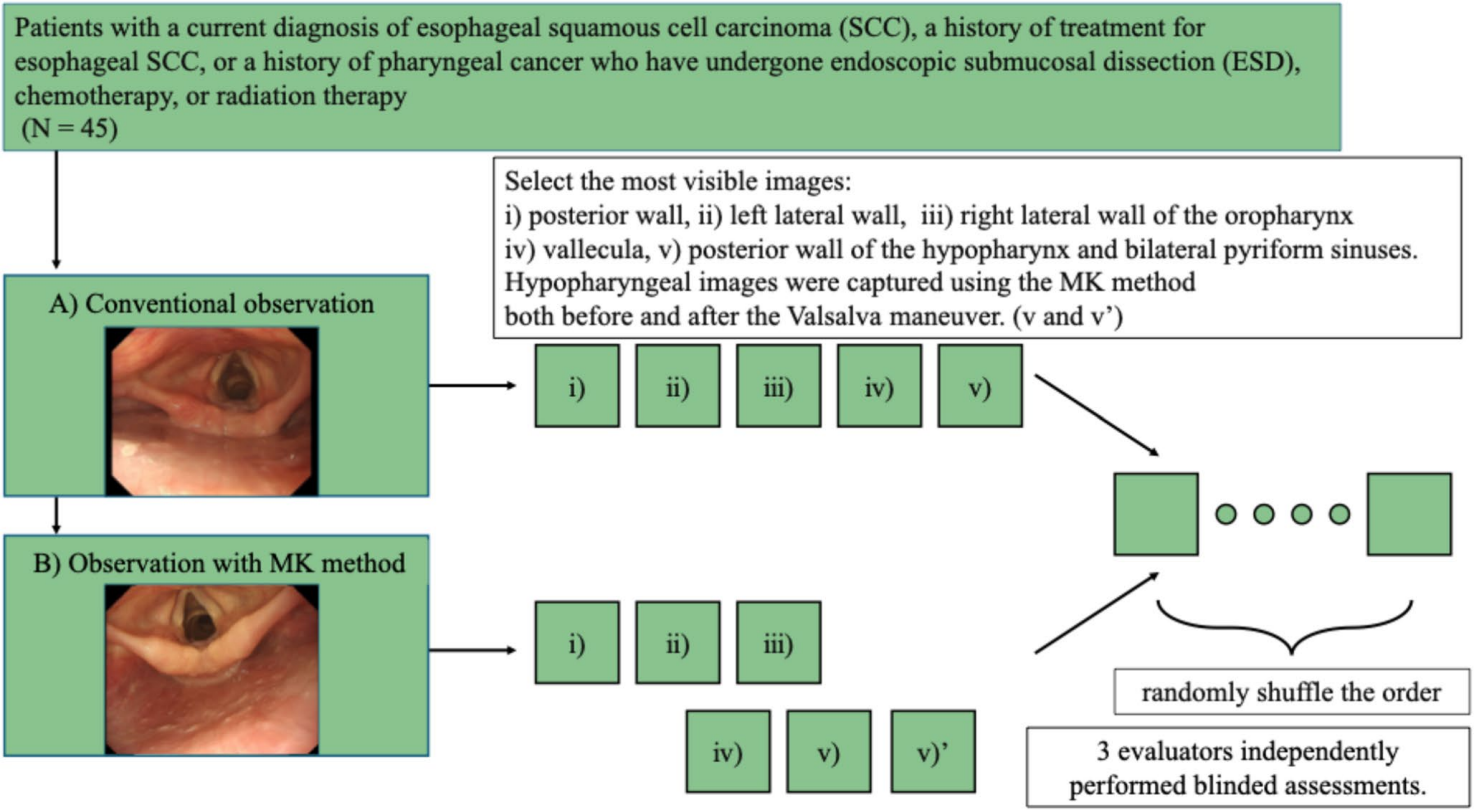
Comparison and evaluation process of conventional and Modified Killian (MK) methods in pharyngeal endoscopy. Pharyngeal observation was performed using two methods: (1) Conventional method (left lateral decubitus position). (2) MK method (Modified Killian position). For the hypopharynx, the MK method was further assessed under two conditions depending on the presence or absence of the Valsalva maneuver. Thus, three observation conditions were analyzed: Conventional method (without Valsalva), MK method without Valsalva, MK method

Following the examinations, the endoscopist reviewed the captured endoscopic images and selected a representative static image demonstrating the best-achieved view for each predefined anatomical site. For hypopharyngeal assessment using the MK method, images were selected before and after the Valsalva maneuver, resulting in two images for this site under this condition. This yielded five images per patient for the conventional method and six images for the MK method, totaling 11 images per patient. Identifiers were removed, and all images were anonymized. For each patient, images obtained under different observation conditions were randomly reordered for presentation to the evaluators (Fig. 1).

### Evaluation process and endpoints

**Fig. 2:**
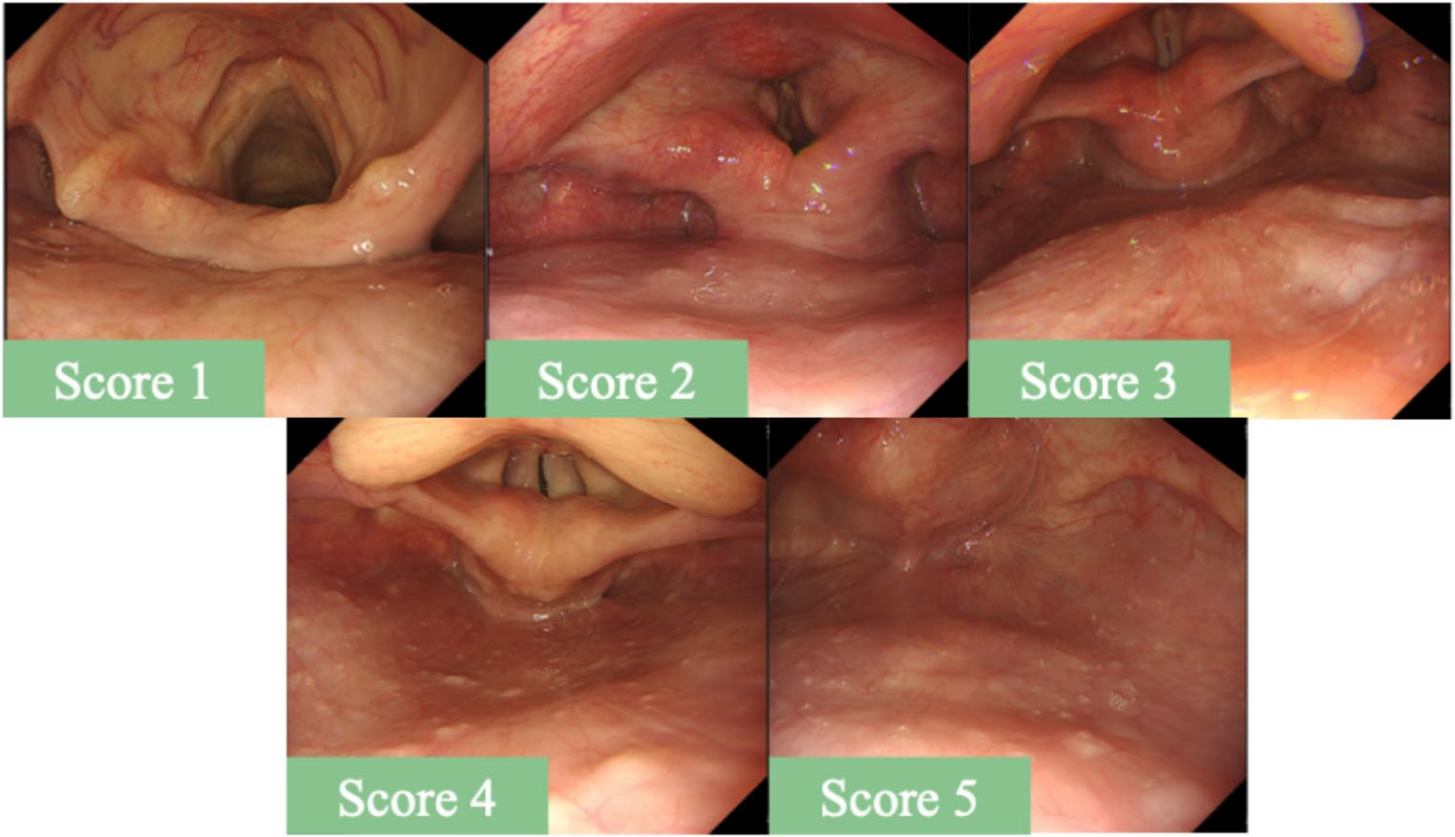
Representative endoscopic images for the hypopharyngeal visibility scoring system. The visibility is scored from 1 to 5 based on the visualization of key anatomical landmarks: pyriform sinuses (PS), postcricoid area (PC), posterior wall (PW), and upper esophageal sphincter (UES). **A** Score 1: Only the bilateral PS are visible; the postcricoid region is collapsed. **B** Score 2: The PC is partially visible but remains in contact with the PW. **C** Score 3: The entire PC is visible, and a distinct space is observed between the PC and the PW. **D** Score 4: A wider view extending beyond the PC is achieved, with a wide space between the PC and PW, but the UES is not yet clearly identified. **E** Score 5: A continuous view extends to the esophageal orifice, and the UES is clearly observed. *PS* Pyriform sinus, *PC* Postcricoid area, *PW* Posterior hypopharyngeal wall, *UES* Upper esophageal sphincter

Three evaluators (gastrointestinal endoscopists with 10, 13, and 19 years of experience), independent of the performing endoscopists, assessed all anonymized and randomized images. Before evaluation, the three evaluators conducted a joint training and calibration session using sample images (Fig. 2). The primary endpoint was the comparison of hypopharyngeal visibility scores among the three observation conditions. Hypopharyngeal visibility was scored on a 5-point scale from 1 (pyriform sinuses only) to 5 (clear view of the upper esophageal sphincter [UES]), modified from previous studies [9, 12–14]. The scoring criteria were as follows: 1, only the pyriform sinuses were observed; 2, the post-cricoid (PC) area was partially observed but was still in contact with the PW; 3, the entire PC area was observed, and a space between the PC area and PW was visible; 4, an area wider than the entire PC area was observed, and the space between the PC area and PW was wide, but the UES was not yet clearly identified; 5, the UES was clearly observed. The hypopharyngeal visibility score was independently assessed for each condition for each patient by the three evaluators; the median was used as the representative value for comparison. Secondary endpoints involved comparing the visibility of other pharyngeal areas (posterior oropharyngeal wall, left lateral wall, right lateral wall, and valleculae), total procedure time, detection rate of new pharyngeal lesions, and incidence of procedure-related adverse events. The visibility of other pharyngeal areas was assessed dichotomously (visible/not visible). Total procedure time was defined as the duration from endoscope insertion into the nasal cavity to its withdrawal. Additionally, to verify the efficacy of the MK method across diverse patient profiles, we stratified hypopharyngeal visibility scores using the three methods according to factors potentially impacting visualization. These factors included prior treatments (e.g., pharyngeal/laryngeal ESD or radiation therapy, esophageal radiation) and key baseline characteristics, such as older age (≥ 75 years), obesity (body mass index ≥ 25 kg/m^2^) [15], male sex, heavy alcohol intake (≥ 280 g/week) [16], heavy smoking (pack-years ≥ 20) [17], and a sensitive gag reflex.

### Statistical analysis

Analyses were performed using R software (v.4.2.0). Continuous variables are summarized as median and interquartile range (IQR) and categorical variables as counts and percentages. The primary endpoint—the visibility scores—was compared using the Friedman test. Post-hoc pairwise comparisons (conventional vs. MK without Valsalva; conventional vs. MK) were performed using the Wilcoxon signed-rank test with a Bonferroni-corrected significance level of *p* < 0.025. For other analyses, *p* < 0.05 was considered significant. Visibility of other pharyngeal areas was compared using McNemar’s test. Interobserver agreement among the three evaluators for the hypopharyngeal visibility score was assessed using the intraclass correlation coefficient (ICC) with a two-way random-effects model for single measures and absolute agreement [ICC (2, 1)].

## Results

### Patient characteristics, pharyngeal visualization, lesion detection, procedure time, and safety

Table 1 presents the patients’ baseline characteristics. The distribution of the hypopharyngeal visibility scores for each method is detailed in Table 2. There was a significant difference in the scores among the three observation conditions (*p* < 0.001). Post-hoc pairwise comparisons with Bonferroni correction demonstrated that the MK method without the Valsalva maneuver yielded significantly higher hypopharyngeal visibility scores than the conventional method (*p* < 0.001). The MK method with the Valsalva maneuver further improved the visibility (*p* < 0.001 vs. conventional). No significant differences were observed between the MK and conventional methods for the visibility of the posterior oropharyngeal wall, left lateral wall, right lateral wall, or valleculae (Table 2). The median procedure time was significantly longer for the MK method (237 s [IQR: 180–267]) than for the conventional method (134 s [IQR: 103–162]) (*p* < 0.001).

**Table 1:** Baseline patient characteristics (N = 45)

Characteristic	Value
Age, median [IQR]	73 [67–77]
ECOG performance status, n (%)	
0	45 (100%)
BMI, kg/m², median [IQR]	21.1 [19.9–22.6]
Sex, n (%)	
Male	39 (86.7%)
Female	6 (13.3%)
History of cancer and treatment, n (%)	
Esophageal cancer	43 (95.6%)
Esophageal operation	12 (26.7%)
Esophageal chemo/radiotherapy	5 (11.1%)
Esophageal ESD	23 (51.1%)
Pharyngeal cancer	13 (28.9%)
Pharyngeal chemo/radiotherapy	4 (8.9%)
Pharyngeal ESD	8 (17.8%)
Alcohol habit	
Alcohol intake, g/week, median [IQR]	331.8 [245.0–497.0]
Flushing history, n (%)^†^	23 (51.1%)
Smoking habit	
Current or former smoker, n (%)	39 (86.7%)
Pack-years, median [IQR]^‡^	30.0 [7.5–50.0]
Gag reflex sensitivity present, n (%)^§^	10 (22.2%)

*IQR* Interquartile range, *ECOG* Eastern cooperative oncology group, *ESD* Endoscopic submucosal dissection, *BMI* Body mass index

^†^Assessed based on patient responses to a pre-examination questionnaire regarding facial flushing after alcohol consumption

^‡^Calculated as (number of cigarettes smoked per day × number of years smoked)/20

^§^Assessed based on patient responses to a pre-examination questionnaire regarding gag reflex sensitivity

Three lesions that were not detected using the conventional method were identified using the MK method. Online Resource 2 demonstrates the detection process of one such lesion using the MK method. Biopsies were performed on the three lesions; they were diagnosed as intraepithelial carcinomas. No adverse events were observed with the conventional or the MK method.

There were no cases in which the MK method itself could not be performed; all 45 patients were able to assume the seated position and adopt the MK posture (neck flexion with chin tucked and head rotation). However, some patients had difficulty performing the Valsalva maneuver adequately, which may have contributed to suboptimal visualization in certain cases. No patient requested procedure discontinuation.

**Table 2:** Comparison of primary and secondary outcomes between the conventional method, the MK method without Valsalva maneuver, and the MK method (N = 45)

Characteristic	Conventional method (*n* = 45)	MK method without Valsalva (*n* = 45)	MK method (*n* = 45)	*p*-value
Hypopharyngeal visibility score				< 0.001^†^
Median [IQR]	1.0 [1.0–2.0]	2.0 [2.0–4.0]	4.0 [3.0–5.0]	
Score distribution, n (%)				
Score 5	0 (0.0)	3 (6.7)	15 (33.3)	
Score 4	3 (6.7)	11 (24.4)	18 (40.0)	
Score 3	6 (13.3)	5 (11.1)	6 (13.3)	
Score 2	10 (22.2)	23 (51.1)	6 (13.3)	
Score 1	26 (57.8)	3 (6.7)	0 (0.0)	
Pairwise comparison vs. conventional	Reference	< 0.001^‡^	< 0.001^‡^	N/A
Visibility of other areas^§^, n/N (%)				
Posterior oropharyngeal wall	40/45 (88.8)	N/A	41/45 (91.1)	> 0.99
Left lateral wall	40/45 (88.8)	N/A	41/45 (91.1)	> 0.99
Right lateral wall	40/45 (88.8)	N/A	42/45 (93.3)	0.50
Valleculae	37/45 (82.2)	N/A	38/45 (84.4)	> 0.99
Procedure time, seconds				
Median [IQR]	134 [103–162]	N/A	237 [180–267]	< 0.001^¶^
Detection of new lesions, n/N (%)	0/45 (0.0)	N/A	3/45 (6.7)	
Adverse events, n/N (%)	0/45 (0.0)	N/A	0/45 (0.0)	N/A

Visibility score for the hypopharynx (range 1–5): 1, Only pyriform sinuses observed; 2, Post-cricoid (PC) area partially observed; 3, PC area entirely observed with space visible; 4, Wider area than PC area observed; 5, Upper esophageal sphincter clearly observed

*IQR* Interquartile range, *MK* Modified Killian, *N/A* Not applicable or not assessed in this comparison

^†^Friedman test for overall comparison among the three methods

^‡^Wilcoxon signed-rank test with Bonferroni correction for two pre-specified pairwise comparisons against the conventional method. Adjusted significance level *p* < 0.025

^§^Dichotomously assessed (visible/not visible). Comparison between conventional and the MK method with Valsalva using McNemar’s test

^¶^Wilcoxon signed-rank test

### Interobserver agreement

The interobserver agreement among the three evaluators for the hypopharyngeal visibility score was good (ICC [2, 1] = 0.863, 95% CI: 0.824–0.896). The mean pairwise weighted kappa (quadratic weights) was 0.862 (range: 0.835–0.916), indicating almost perfect agreement.

### Improvement in the hypopharyngeal visibility using the MK method in patient subgroups

Table 3 details the hypopharyngeal visibility scores achieved with the three methods, stratified according to treatment history and baseline characteristics. Prior treatment history, including ESD or radiation therapy involving the pharynx, larynx, or esophagus, is often associated with post-treatment anatomical changes or scarring that hinder visualization. Despite these challenges, the MK method yielded improved visibility scores in these subgroups, including in the patients with a history of pharyngeal/laryngeal ESD (*n* = 8) and radiation therapy (*n* = 4). Furthermore, consistent improvements in visibility were observed across all stratified subgroups.

**Table 3:** Comparison of hypopharyngeal visibility scores between the conventional and Modified Killian methods stratified by background factors

Patient subgroup	*n*	Conventional method (median [IQR])	MK method without Valsalva (median [IQR])	MK method (median [IQR])
Older age (≥ 75 years)	16	1.5 [1.0–2.5]	2.0 [2.0–3.2]	4.0 [3.0–4.5]
Obesity (BMI ≥ 25 kg/m2)	3	2.0 [1.5–2.5]	2.0 [2.0–2.5]	4.0 [3.0–4.0]
Male	39	1.0 [1.0–2.0]	2.0 [2.0–3.5]	4.0 [3.0–5.0]
Heavy alcohol consumption (alcohol intake ≥ 280 g/week)	27	2.0 [1.0–2.0]	2.0 [2.0–4.0]	4.0 [3.5–5.0]
Heavy smoking (pack-years ≥ 20)	30	1.0 [1.0–2.0]	2.0 [2.0–3.8]	4.0 [4.0–5.0]
Sensitive gag reflex	10	1.0 [1.0–2.0]	2.0 [2.0–3.0]	4.0 [4.0–5.0]
History of pharyngeal/laryngeal ESD	8	1.0 [1.0–1.0]	2.0 [2.0–3.2]	3.5 [2.25–5.0]
History of pharyngeal/laryngeal radiation	4	1.5 [1.0–2.0]	2.0 [2.0–2.7]	4.5 [3.5–5.0]
History of esophageal radiation	5	1.0 [1.0–1.0]	2.0 [1.0–2.0]	3.0 [2.0–5.0]

*IQR* Interquartile range, *MK* Modified Killian, *ESD* Endoscopic submucosal dissection, *BMI* Body mass index

## Discussion

The hypopharyngeal visibility achieved via the MK method was found to be significantly better than that achieved using the conventional approach. Notably, the MK method without the Valsalva maneuver—reflecting the independent effect of the MK position itself—yielded significantly higher visibility scores than the conventional method, and the addition of the Valsalva maneuver further improved the visualization, indicating that the positional component and the Valsalva maneuver have additive effects, with each contributing independently. Importantly, the MK method enabled the detection of additional tumors in 6.7% of patients (3/45). Despite the longer procedure time, no adverse events occurred, suggesting that the MK method can be safely applied in high-risk patients. Furthermore, consistent improvements in visibility were observed across various subgroups, including in patients with prior treatments, older age, obesity, history of heavy alcohol or tobacco use, and sensitive gag reflexes, demonstrating the robustness and generalizability of this method. However, the small number of patients in each subgroup limits the statistical power to detect differential benefits, and further studies with larger sample sizes are needed to identify specific patient populations that may benefit most from this technique.

The fundamental principle enabling this visual expansion lies in the specific head positioning and its anatomical effects. In the MK method, the patient maintains a forward flexion posture with the chin firmly tucked in while rotating the head. This maneuver physically widens the space between the PC area and the PW, creating a favorable field of view. Furthermore, the method allows for dynamic adjustment; if an ideal view is not obtained with a single movement, appropriate additional instructions can be provided to shift the head position until optimal visualization is achieved. This simplicity of the maneuver—relying on basic body mechanics rather than complex instrumental manipulation—ensures high reproducibility among different endoscopists, facilitating its widespread adoption. Additionally, the epiglottic vallecula can also be easily observed by performing neck extension, the reverse of the standard MK position.

Hypopharyngeal observation is often more difficult in patients who have undergone prior pharyngeal, laryngeal, or esophageal treatments, such as ESD or radiation, owing to anatomical changes; however, the MK method improved visibility even in such cases. Although the inclusion of patients who had undergone prior pharyngolaryngeal cancer treatment introduced clinical heterogeneity, these patients were among the highest-risk groups for developing metachronous cancers due to field cancerization, necessitating continued surveillance. Post-treatment scarring and anatomical changes often make hypopharyngeal visualization particularly challenging, which is precisely the clinical scenario wherein the MK method may provide the greatest benefit. The prevalence of second primary head and neck cancers in patients with ESCC is 6.7%, with 60% of these occurring in the hypopharynx [18]. To reduce the rate of missed HNSCC, it is crucial to actively use image-enhanced endoscopy (IEE) techniques such as NBI and blue laser imaging [4, 19, 20]. Muto et al. revealed that IEE improved the detection rate of superficial squamous cell carcinoma from 55% to 97% in the esophagus and from 8% to 100% in the pharynx [21]. However, when observing the hypopharyngeal lumen, a limited field of view can hinder lesion detection; therefore, improvement cannot be achieved using IEE alone. This limitation was addressed in our study as the conventional method yielded adequate visualization (score ≥ 4) in only 6.7% of cases; the MK method without the Valsalva maneuver, in 31.1% of cases; and the MK method with the Valsalva maneuver, in 73.3% of cases, indicating the superiority of the MK method. Beyond mere detection, the MK method proved to be highly beneficial for precise lesion boundary delineation, which is clinically pivotal given the anatomical characteristics of the hypopharynx (thin mucosa and narrow margin between a resectable early lesion and an invasive cancer requiring surgery). Accurate delineation allows for the application of minimally invasive treatments such as ESD, contributing directly to organ preservation.

Although this study was limited to transnasal endoscopy, magnifying endoscopy plays an important role in esophageal cancer surveillance. However, regarding pharyngeal observation, oral endoscopes with magnifying capability have a larger diameter, making pharyngeal insertion more difficult and causing greater patient discomfort compared with that experienced during transnasal endoscopy. We believe that the two approaches—magnifying endoscopy for the esophagus and transnasal endoscopy combined with the MK method for the pharynx—can be used in a complementary manner, each optimized for its respective target site.

The Valsalva maneuver is a well-known method for observing the hypopharynx. Several methods have been reported for the Valsalva maneuver in transoral endoscopy [11–14, 22]. In the transnasal endoscopy setting used in our study, the Valsalva maneuver can be performed without a dedicated mouthpiece and in the left lateral decubitus position, which is a notable advantage [22]. Although the Valsalva maneuver is a well-known technique for expanding the hypopharynx, it has drawbacks. Its success relies heavily on the ability of the patient to coordinate breathing (risk of failure to hold breath) and is often operator-dependent, resulting in transient views. In contrast, our results showed that the MK method provides stable and sustained visibility through physical repositioning of the head and neck, even without the Valsalva maneuver. We consider the MK method and the Valsalva maneuver to be complementary rather than mutually exclusive, and their combination yielded the best results in our study (median score 4.0). A direct comparison between the MK method and the transnasal Valsalva maneuver alone remains an important future research question.

This study has some limitations. First, its retrospective nature and relatively small sample size limit the generalizability of its findings. A formal a priori sample size calculation was not performed, and this may be considered as an exploratory study. Moreover, to the best of our knowledge, no prior studies on the MK method in gastrointestinal endoscopy have provided sufficient data for a robust sample size estimation. Therefore, future prospective studies incorporating an appropriate sample size calculation based on the effect sizes and variance observed in the present study are warranted. Second, the sequence of examinations was fixed, with conventional observation always preceding the MK method, possibly introducing a bias. This examination-order bias is bidirectional: the second examination (MK method) may have been disadvantaged by patient fatigue and accumulated gag reflex sensitivity; however, it may also have benefitted from familiarity effects for both the patients and endoscopists, as well as easier nasal reinsertion. These opposing effects cannot be fully eliminated from our study design. Future prospective studies should employ a randomized crossover design to eliminate these confounding factors. Third, patient comfort and tolerability were not systematically assessed, although participants were informed that they could request discontinuation, and none did so. Fourth, although external evaluators were independent of the performing endoscopists and underwent standardization, image selection was operator-dependent and may have introduced selection bias. Fifth, scoring was based on subjective evaluation, and although reproducibility was supported by training, some interobserver variation may have remained. Sixth, the inclusion of patients who had undergone prior pharyngolaryngeal cancer treatment may have introduced heterogeneity; nonetheless, given that they were at a high risk of metachronous cancers, we consider their inclusion clinically appropriate. Finally, there may be residual selection bias, although all consecutive clinically relevant cases were included in the study and no patients were arbitrarily excluded.

Despite these limitations, the MK method demonstrated significant potential for enhancing hypopharyngeal visualization safely and effectively. Future prospective randomized trials are warranted to validate whether this improved visibility translates into higher detection rates of early-stage lesions and systematically assess patient tolerability.

In conclusion, our findings suggest that the MK method is a feasible and effective option for routine screening in populations at high risk of head and neck malignancies.

## Supplementary Information

Below is the link to the electronic supplementary material.

Supplementary Material 1

Supplementary Material 2
